# Analysis of Overall Survival in Patients With Multiple Primary Malignancies: A Single-center Experience

**DOI:** 10.7759/cureus.4552

**Published:** 2019-04-27

**Authors:** William P Skelton, Azka Ali, Michelle N Skelton, Roland Federico, Raphael Bosse, Thu-Cuc Nguyen, Long H Dang, Rohit Bishnoi

**Affiliations:** 1 Internal Medicine, University of Florida, Gainesville, USA; 2 Miscellaneous, Columbia University, New York, USA; 3 Internal Medicine, University of Central Florida, Orlando, USA; 4 Oncology, University of Florida, Gainesville, USA; 5 Hematology and Oncology, University of Florida, Gainesville, USA

**Keywords:** multiple primary malignancy, multiple primary malignancies, multiple primary malignant neoplasms, multiple primary cancers

## Abstract

Introduction

Multiple primary malignancies (MPMs) are seen in ~5% of all tumors. The aim of this study was to determine the quantitative impact on overall survival (OS) and treatment choices in patients with MPMs.

Methods

A retrospective analysis to determine patients with MPMs was conducted over a six-year period. Patients were defined as simultaneous MPMs if the second malignancy was discovered within 60 days of the first, and as sequential MPMs if discovered after 60 days of the first.

Results

Fifty-six patients with MPMs as defined above were identified, 38 (68%) simultaneous and 18 (32%) sequential. Development of second malignancy did not affect treatment in 47 (84%) of patients. Median OS after diagnosis of first malignancy was 13.0 months (95% confidence interval (CI) 10.3-15.8 months), compared to 10.6 months (95% CI 7.1-13.9 months) after the diagnosis of second malignancy. Median OS for the simultaneous MPM group was 13.5 months (95% CI 7.1-19.9 months), compared to 3.2 months (95% CI 0.0-9.8 months) for the sequential MPM group.

Conclusions

The development of a second malignancy impacts OS and treatment decisions. Patients who developed sequential MPM performed poorer than those who developed simultaneous MPM. This was likely in part due to effects of existing treatment on performance status as well as treatment preferences when second MPM is diagnosed (as many patients opted for supportive care after second MPM). Further analysis with larger patient cohorts is necessary to ascertain the aforementioned effects of OS and treatment options with respect to tumor pathology, stage, and performance status.

## Introduction

Multiple primary malignancies (MPMs) are defined as two or more malignancies arising independently of one another in the same or different organs, while excluding metastatic sites of the primary malignancy. This term was initially defined by Warren and Gates in 1932 [1]. MPMs are further divided into synchronous and metachronous malignancies. The synchronous group is defined as the diagnosis of a second malignancy within six months of initial primary, and the metachronous group is defined as diagnosis after six months of initial primary [2]. It is important for the clinician to distinguish between MPMs and metastatic spread, as this has impacts on both prognosis and subsequent treatment.

MPMs are not a rare phenomenon and the incidence is estimated at up to 5% in all tumors [3]; and estimated at 8% by the National Cancer Institute (NCI)’s Surveillance, Epidemiology, and End Results (SEER) data [4]. Not surprisingly due to its prevalence, lung cancer is the most commonly diagnosed malignancy in patients with MPMs. The incidence of diagnosis of MPMs is increasing secondary to multiple reasons, including but not limited to improved diagnostic techniques as well as novel treatment modalities leading to prolonged survival in patients with malignancies [5]. With life expectancy predicted to continue to increase over the coming years [6], so too is the expected incidence of malignancy and likely also patients with MPMs. The incidence of patients with MPMs with more than two malignancies was found to be 0.1% in an analysis of over 50,000 patients over 40 years [5], and 2%-12% of that subset of patients with two or more MPMs develop additional MPMs during their disease course [5].

Kim et al. showed that advanced lung cancer was an independent poor prognostic factor in both synchronous and metachronous groups. In the metachronous group, head and neck cancer as an MPM was a poor prognostic factor [2]. Other studies have demonstrated smoking as a risk factor for MPMs. Digestive tract, colorectal and cervical cancers have been shown as the most frequent cancers to be MPMs along with lung cancers [6]. Environmental exposures and germline mutations have also led to a certain pattern of associated MPMs, including lung/head and neck cancers [7], and breast/colorectal cancers [8]. A subgroup analysis also showed that patients with multiple malignancies had better survival than the general cancer population [6]. This may be in part from closer follow up and more intense therapies, but there it is possible that multiple malignancies compete for sites of metastasis and thereby control one another’s spread. Multiple theories regarding atypical metastases and MPMs have been proposed. Currently postulated theories include a) MPMs may arise from cancer-initiating cells (CICs) mutated at an early age of embryogenesis, or b) MPMs may develop from different CICs comprising the term “multigenesis [9]."

In our clinical observation, we observed that patients developing second malignancy while they are being treated for first malignancy tend to do poorly as compared to other patients that have only one active malignancy. Developing second malignancy makes treatment decisions more challenging as now treatment choices are made considering both active malignancies. In our clinical practice, we have noticed that rarely second malignancy is discovered incidentally while patients are being worked up for first malignancy. In other cases, second malignancy develops de-novo while patients are being treated for first. The aim of this retrospective study was to find the quantitative impact on overall survival (OS) of two different malignancies at our institution.

## Materials and methods

This single-center retrospective study was conducted at the University of Florida (UF) Cancer Center. The study population was identified through institutional tumor cancer registry, institutional electronic medical record (EMR), as well as the use of ICD-9 and ICD-10 codes for cancer diagnoses. This study was approved by the UF Institutional Review Board (IRB); IRB#201701157. The time span included a six-year period from July 1, 2011 to June 30, 2017. Inclusion criteria included patients with diagnosis of two or more concurrent malignancies on active treatment for their cancers. Exclusion criteria included patients with two or more cancers in the same organ system as well as slow-growing cancers such as skin cancers.

Patients were defined as simultaneous MPMs if the second malignancy was discovered within 60 days of the first, and defined as sequential MPMs if discovered after 60 days of the first. We chose a 60-day time frame as most patients complete their staging work up in this time frame, and occult second malignancy was felt likely to be identified during this time of staging. Data was collected from EMR individual chart review and available through the cancer registry. Over 1400 patients were preselected on our initial query; once inclusion and exclusion criteria were applied, 56 patients were eligible to be included in the study. Data was analyzed for patient characteristics, tumor pathology and treatments received. Kaplan Meier survival analysis was performed to estimate overall survival using Statistical Package for the Social Sciences (SPSS) version 25 (SPSS Inc., Chicago, IL, USA). Descriptive data were presented as number and percentages. Data was analyzed using Breslow-Day and log-rank methods. A p-value < 0.05 was considered statistically significant.

## Results

A total of 56 patients with MPMs as defined above were identified, 38 (68%) simultaneously diagnosed and 18 (32%) with sequential diagnoses. Forty-seven patients (84%) were male, 48 (86%) were white, and 43 (77%) were active or former smokers (Table 1).

**Table 1:** Demographics of patients with multiple primary malignancies

Variables		n	%
Gender	Male	47	83.9
	Female	9	16.1
Race	White	48	85.7
	Black	5	8.9
	Unknown	3	5.4
Timing of Second Malignancy Diagnosis	Simultaneous	38	67.9
	Sequential	18	32.1
Smoking Status	Never smoked	12	21.4
	Remote smoker	29	51.8
	Active smoker	14	25.0
	Unknown	1	1.8

Malignancies of the head and neck region (n=11, 19.6%) and gastrointestinal (GI) tract (n=9, 16.1%) were the most common sites where first malignancies were found, and these same two tumor sites were the most common sites for secondary malignancies. It was noted that incidence of renal cell cancer (n=8, 14.3%) and lymphoma (n=5, 8.9%) were higher as secondary malignancies than their incidence as first malignancy. Twenty patients (35.7%) had their second malignancy within the same organ system (Tables 2-4).

**Table 2:** Sites of first and second malignancies

Organ system	Malignancy type	First malignancy		Second malignancy	
		n	%	n	%
Genitourinary	Renal cell cancer	4	7.1	8	14.3
	Prostate	3	5.4	2	3.6
	Other	0	0.0	3	5.4
Lungs	Small cell	1	1.8	2	3.6
	Non-small cell	5	8.9	7	12.5
	Unknown	2	3.6	0	0.0
Gastrointestinal	GI tract	9	16.1	9	16.1
	Hepatobiliary	3	5.4	3	5.4
	Pancreas	1	1.8	2	3.6
Head and Neck	HNSCC	11	19.6	9	16.1
	Parotid	1	1.8	0	0.0
	Thyroid	0	0.0	1	1.8
Blood and lymphatics	Myeloma	5	8.9	0	0.0
	Leukemia	6	10.7	2	3.6
	Lymphoma	3	5.4	5	8.9
	Others	1	1.8	2	3.6
Other	Sarcoma	1	1.8	1	1.8

**Table 3:** Distribution of malignancies if both multiple primary malignancies (MPMs) were from the same organ system

Organ system	n	%
Genitourinary	3	5.4
Blood/Bone marrow	2	3.6
Respiratory	1	1.8
Gastrointestinal	7	12.5
Head and Neck	5	8.9
Lymphatic	2	3.6

**Table 4:** General stage of malignancy

General stage	First malignancy		Second malignancy	
	n	%	n	%
LOCAL	17	30.4	23	41.1
REGIONAL	14	25.0	12	21.4
DISTANT	21	37.5	15	26.8
UNKNOWN	4	7.1	6	10.7

Development of second malignancy did not alter or affect the treatment of first malignancy in 47 patients (83.9%). Nine patients (16.1%) were treated with best supportive care for first malignancy while 20 patients (35.7%) were treated with best supportive care of second malignancy (Table 5). 

**Table 5:** Distribution of various treatment modalities for first and second malignancies

Treatment modality	First malignancy		Second malignancy	
	n	%	n	%
Chemotherapy	15	26.8	11	19.6
Chemo-Radiation	16	28.6	12	21.4
Chemoembolization	1	1.8	0	0.0
Surgery	4	7.1	5	8.9
Surgery, chemo and radiation	1	1.8	3	5.4
Surgery-Radiation	2	3.6	1	1.8
Radiation	6	10.7	4	7.1
Radiation-Hormonal	1	1.8	0	0.0
Chemo-radiation and hormonal	1	1.8	0	0.0
Best supportive care	9	16.1	20	35.7

**Figure 1:**
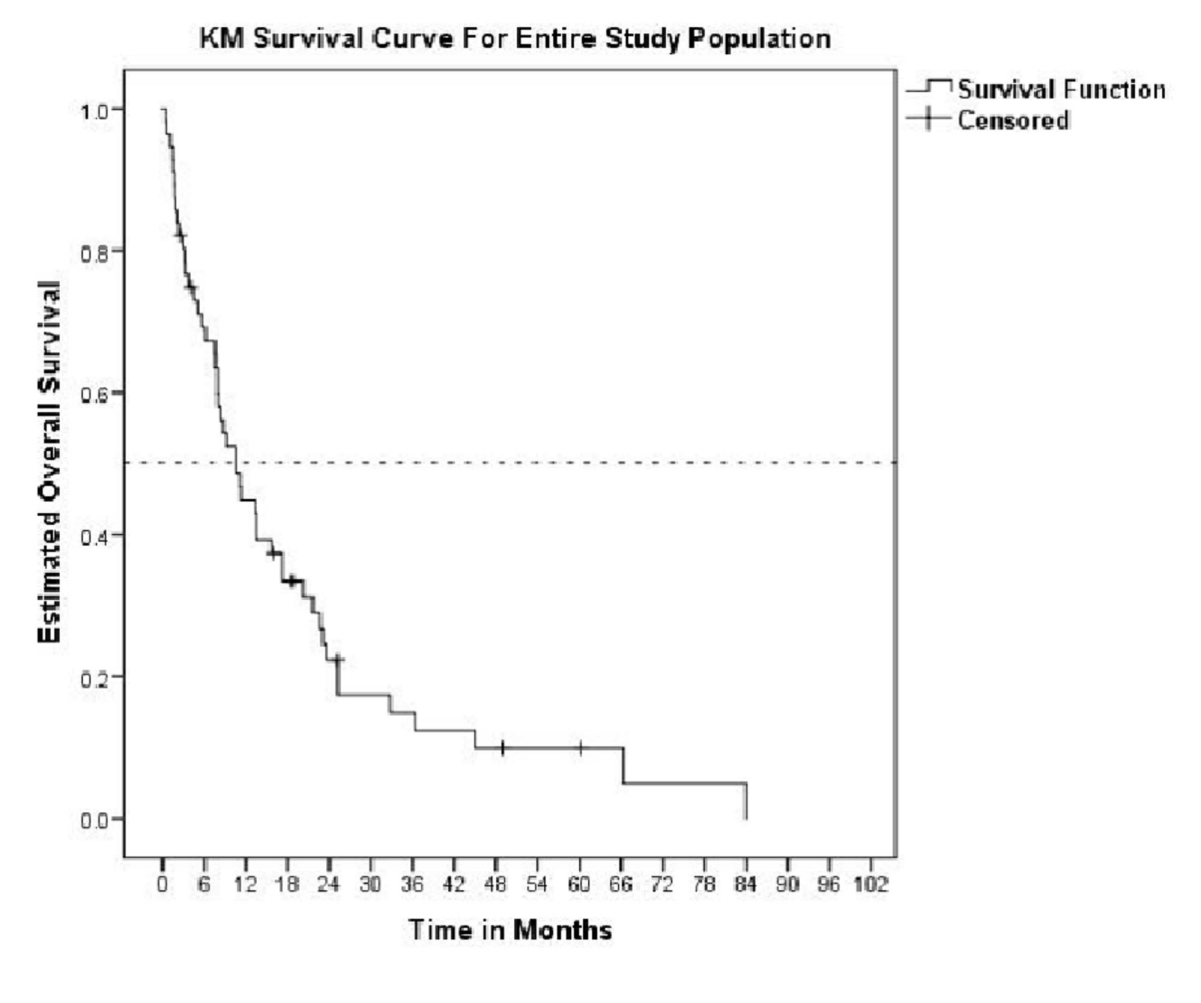
KM curve of the entire study population after diagnosis of second malignancy KM: Kaplan-Meier

**Figure 2:**
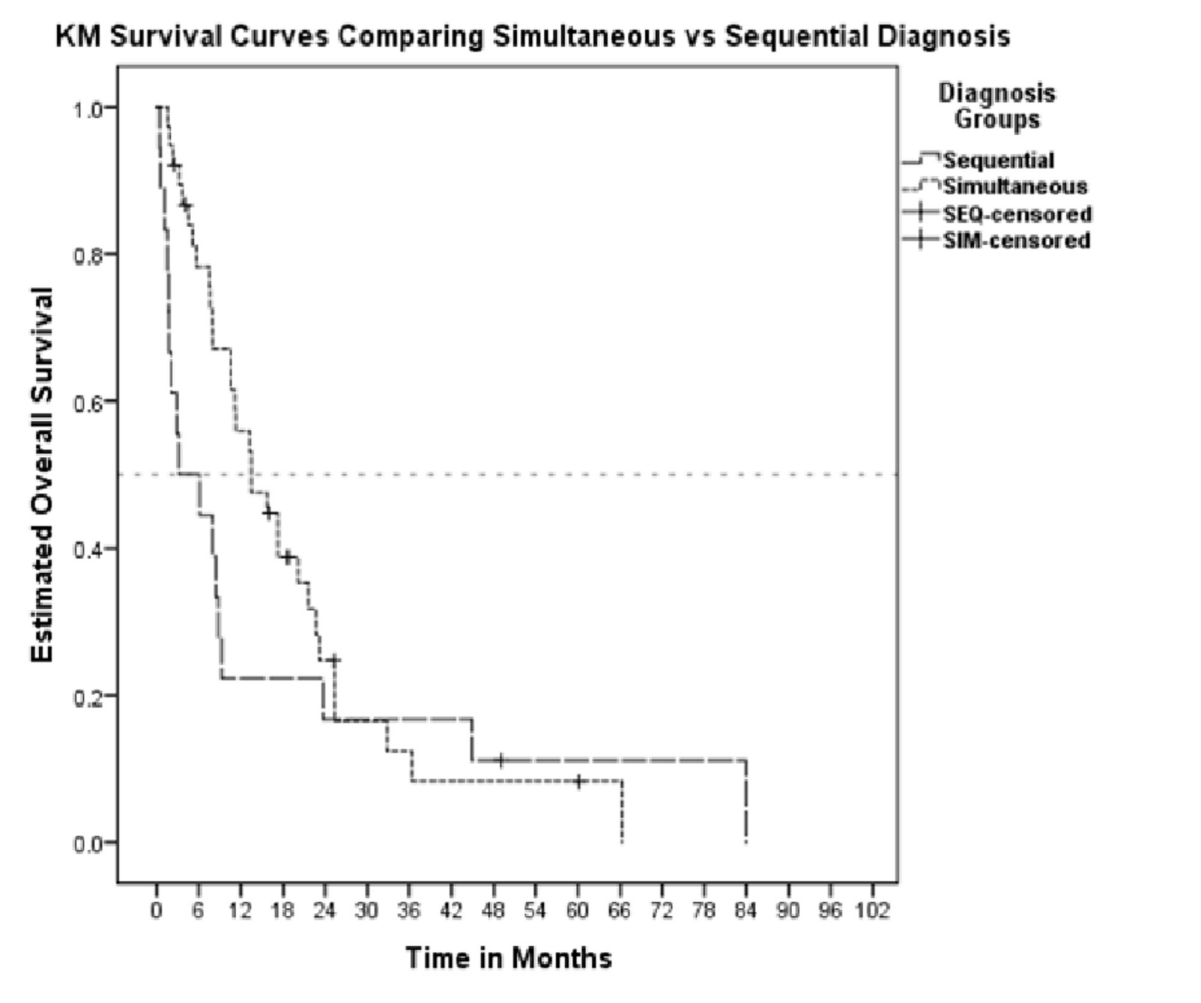
Comparative KM curves for patients with simultaneous vs. sequential diagnosis from date of second diagnosis KM: Kaplan-Meier; SEQ: sequential; SIM: simultaneous.

Forty-eight patients (86%) were deceased at the end of the study period. Median overall survival (OS) after diagnosis of first malignancy was 13.0 months (95% CI 10.3-15.8 months), compared to 10.6 months (95% CI 7.1-13.9 months) after the diagnosis of a second malignancy (p=0.523). Median OS for the simultaneous MPM group was 13.5 months (95% CI 7.1-19.9 months), compared to 3.2 months (95% CI 0.0-9.8 months) for the sequential MPM group (p=0.005) (Figures 1-2).

## Discussion

It has been shown that synchronous malignancies have statistically significant reduction in OS when compared to metachronous malignancies [3], but the quantitative mortality difference is unclear when analyzing simultaneous versus sequential malignancies. We analyzed the quantitative difference in mortality in the latter group to determine the clinically significant difference in OS if malignancy occurs within or after 60 days.

Given our small sample size and the complexity and number of variables, there were few statistically significant differences between different categories (whether simultaneous or synchronous, type of first malignancy or type of second malignancy, type of treatment, etc.) that were found. Analysis regarding the possible biologic basis for the occurrence of MPMs, as well as any clear tendency for multiple malignancies to occur together, thus, at this point, is unfortunately largely speculation.

We did note statistically significant differences in several patient characteristics: 47 patients (84%) were male, 48 (86%) were white, and 43 (77%) were active or former smokers. Patients with a history of tobacco abuse developed MPMs is expected. The fact that a majority of patients found to have MPMs were Caucasian males could be explained by several factors. Caucasian males may have been more inclined to smoke, although their representation within the study still out-numbers even the absolute percentage of total smokers. Given the limitations of a single institutional study, Caucasian males may simply represent a disproportionate amount of our patient population.

Several mutations may be involved in playing a role in patients susceptible to more than one malignancy. Epidermal growth factor receptor (EGFR) mutation, with exon 19 deletion, was seen more commonly in patients with adenocarcinoma of the lung and MPMs [10]. The tumor suppressor gene p53 is the most commonly mutated gene in human cancers. This loss-of-function mutation leads to cell proliferation, impairment of cell cycle checkpoints and arrest, and promotes oncogenesis. One of the largest studies analyzing the frequency of p53 mutations showed that in colorectal cancer, 50% of patients with p53 mutation had one or more pathogenic gene mutations; 47% with non-small cell lung cancer (NSCLC) had one or more pathogenic gene mutations, and 52% of glioma patients had at least one or more pathogenic gene mutations [11]. It must be noted that even germline variants of p53 have different consequences in terms of cancer development and penetrance [12], and hence is not an independent factor in predicting susceptibility to the development of one or more malignancies.

In some cases, there is clearly a temporal association between cancers, for example in the case of lung and upper aerodigestive cancers, although no statistically significant difference in survival was shown in patients with lung cancer and upper aerodigestive cancers (when compared to patients without the latter) [7].

In this study, we show that the second malignancy impacts OS significantly even before six months as shows in the case of synchronous versus metachronous cancers [3]. Patients who developed MPMs in greater than 60 days (sequential group) had a statistically significant reduction in OS when compared to patients who developed MPM in less than 60 days.

Several factors may play a role here. Patients diagnosed with a malignancy (i.e., first malignancy) have frequent follow up in the oncology clinic and hence get frequent imaging for both initial staging as well as to analyze the response of treatment to their malignancy. Frequent imaging and follow-up make it more likely to diagnose a second malignancy, and hence makes it more likely to invasively pursue biopsies of atypical lesions, that may be simply watched in surveillance in a non-oncology clinic setting [13]. Early diagnosis of a second malignancy and prompt treatment likely play a role in improved OS in the simultaneous group.

Cancer patients on active chemotherapy are evaluated for their Eastern Cooperative Oncology Group (ECOG) performance status (PS) and if a second malignancy is diagnosed later (more than 60 days after, sequential group), patients may have developed poorer PS due to effects of existing treatment. It is also likely that with the diagnosis of a second malignancy later on in the treatment (as opposed to simultaneous), patients are more likely to opt for supportive care.

We also noticed an increased incidence of renal cell carcinoma (RCC) and lymphoma as second primaries as compared to their incidence as first primary. Prior research by Royle et al. has reported increased incidence of melanoma and other cancers in patients with chronic lymphocytic leukemia (CLL), with the authors postulating that this might be due to chronic immunosuppression caused by CLL [14]. This finding does suggest the role of immunosuppression in development of second malignancy and also due to the discovery of renal mass on increased surveillance which otherwise might be asymptomatic or clinically benign, and not otherwise detected without this increased surveillance.

Our study has several limitations, first and foremost data being limited as inherit to its retrospective design. In addition, with a small sample size and a low median OS for each group analyzed, it is difficult to control for confounders. As mentioned previously in the discussion, the preponderance of our study population were Caucasian males who were active or former smokers, which may be a limitation of our single-center experience.

We feel that the importance of understanding the complexity of clinical decision-making involved in patients with MPMs should not be understated. The ability to offer patients further prognostic information required of their cancer(s) is a frequent and important clinical question, and this study does provide some insight into the specific question of prognosis in patients with MPMs. Further investigations with larger sample sizes or multicenter investigations as well as a more granular exploration of associations between types of cancer is required.

## Conclusions

This study highlights the impact of second MPM on OS, and the timing of such (sequential or simultaneous) has significant prognostic implications. The significant difference in OS between the simultaneous and sequential groups should prompt discussion between the provider and patient regarding the next step in their care. Due to the rarity of MPMs, a small cohort was identified despite an extensive window of chart review. Further analysis with larger patient cohorts is necessary to ascertain the aforementioned effects of OS and treatment options with respect to tumor pathology, stage, and performance status.
